# Sleep architecture and quality of life in comorbid OSA and depression: cross-sectional analysis of the Sydney sleep biobank

**DOI:** 10.1007/s11325-025-03485-y

**Published:** 2025-09-30

**Authors:** Daryl E. C. Y. Chan, Yu Sun Bin, Philip de Chazal, Peter A. Cistulli, Andrew S. L. Chan, John R. Wheatley, Kristina Karaitis, Brendon J. Yee

**Affiliations:** 1 Woolcock Institute of Medical Research, Macquarie University, 2 Innovation Road, Macquarie Park, Sydney, NSW 2113 Australia; 2 Sleep Research Group, Charles Perkins Centre, University of Sydney, Sydney, NSW Australia; 3 Northern Clinical School, Faculty of Medicine and Health, University of Sydney, Sydney, NSW Australia; 4 School of Biomedical Engineering, University of Sydney, Sydney, Australia; 5 Department of Respiratory and Sleep Medicine, Royal North Shore Hospital, Reserve Rd, St Leonards, NSW 2065 Australia; 6 Ludwig Engel Centre for Respiratory Research, The Westmead Institute for Medical Research, Sydney, NSW Australia; 7 Westmead Clinical School, Faculty of Medicine and Health, University of Sydney, Sydney, NSW 2145 Australia; 8 Department of Respiratory and Sleep Medicine, Westmead Hospital, Westmead, Sydney, NSW 2145 Australia; 9 Department of Respiratory and Sleep Medicine, Royal Prince Alfred Hospital, Camperdown, Sydney, NSW 2050 Australia; 10 Central Clinical School, Sydney Medical School, University of Sydney, Sydney, Australia

**Keywords:** Obstructive sleep apnoea, Depression, Sleep architecture, Polysomnogram, Quality of life, Epworth sleepiness scale, FOSQ-10, DASS-21

## Abstract

**Introduction:**

Obstructive sleep apnoea (OSA) and depression are individually associated with changes in sleep architecture and reduced quality of life. However, there are few studies which report on the joint impact of comorbid OSA and depression.

**Methods:**

821 participants from the Sydney Sleep Biobank database were assessed (38% female; age 49.5, SD 15.6 years). Participants were patients who were referred for and underwent an overnight sleep study on suspicion of sleep disordered breathing. Participants were divided into 4 groups based on the apnoea-hypopnoea index and depression score from the Depression Anxiety Stress Scale-21; (1) no OSA-no depression, (2) OSA-only, (3) depression-only, (4) comorbid OSA and depression (OSAD). Sleep architecture and quality of life (Epworth Sleepiness Scale, ESS and Functional Outcomes of Sleep, FOSQ-10) scores were compared between groups. Confounders considered included age, gender, body mass index, alcohol, and psychiatric medications.

**Results:**

Patients with OSAD and depression-only had higher ESS scores (8.4 vs 8.9 vs 6.9, p=0.003) and lower FOSQ-10 scores (13.9 vs 12.8 vs 16.7, p<0.001) than those with OSA-only. However, after control for confounders and excluding patients on psychiatric medications, OSAD was not associated with any significant changes in sleep architecture compared to those with OSA-only.

**Discussion/Conclusions:**

Despite the lack of changes in sleep architecture, it is still important toidentify comorbid OSA and depression as it can be associated with worsequality of life and this may affect treatment compliance.

**Supplementary Information:**

The online version contains supplementary material available at 10.1007/s11325-025-03485-y.

## Introduction

Obstructive sleep apnoea (OSA) is characterised by repetitive upper airway obstruction during sleep, resulting in repetitive oxygen desaturation, increased sympathetic activity, and sleep fragmentation [1]. Patients with OSA may report fatigue, excessive daytime sleepiness, and morning headaches. In addition to daytime sleepiness, OSA is associated with significant health consequences including hypertension, cardiovascular disease, motor vehicle accidents, and depression. Numerous studies have shown an increased risk of depression as a consequence of OSA, and a meta-analysis has shown that OSA doubles the risk of incident depression [2]. 

Depression is a common mental health disorder characterised by low mood and anhedonia, in addition to sleep related symptoms. Up to 90% of patients with depression have a sleep complaint [3]. There is considerable overlap between the symptoms of OSA and depression, with fatigue, impaired concentration, mood disturbances, and irritability being common in both. Treatment of OSA with continuous positive airway pressure (CPAP) for more than 2 months has been shown to improve depressive symptoms [4]. A community study reported that 17% of people with OSA have comorbid depression [5], and the prevalence of depression in OSA patients can be as high as 63% in sleep clinic populations [6, 7]. 

Previous studies have also found that comorbid OSA and depression reduces CPAP adherence [8], results in poorer quality of life [9] and longer and more severe depressive episodes [10]. In addition, recent studies suggest that comorbid OSA and depression is associated with higher cardiovascular mortality and all-cause mortality than OSA or depression alone [11, 12]. Thus identification of comorbid depression in patients with OSA is important as it has a significant impact on patient outcomes.

In addition to clinical and health impacts, each individual disorder has been associated with changes in sleep architecture. OSA reduces proportions of deep sleep (N3 and REM sleep), increases proportions of light sleep (N1), and increases arousals and sleep fragmentation [13, 14]. Patients with depression similarly have a higher proportion of N1 [15, 16] and REM [3], and reduced REM latency [3, 16]. There are few studies reporting on sleep architecture in comorbid OSA and depression, however reported changes include a prolonged REM latency [17], increased proportion of REM [18], and a shorter sleep latency [18].

We aimed to compare sleep architecture and quality of life in comorbid OSA and depression (OSAD) against OSA alone.

## Methods

### Data source

The Sydney Sleep Biobank (SSB) contains data collected from patients undergoing a level 1 diagnostic polysomnogram (PSG) in 3 tertiary hospitals in Sydney, Australia between 2018 and 2023 [19]. Data collected from Biobank participants include sociodemographic information, medical and psychiatric history, regular medications and symptom scores, anthropometric measurements, in addition to polysomnography (PSG).

Biobank data collection was approved by the Northern Sydney Local Health District Human Research Ethics Committee (Ref: HREC/17/HAWKE/340). Ethics approval for the current study was obtained from the University of Sydney Human Research Ethics Committee (Ref: 2024/HE000277). All participants provided written informed consent.

Reporting of this study adhered to the STROBE guidelines for reporting observational studies (Appendix 1) [20]. 

## OSA

All PSGs were visually scored by a trained sleep technician according to the 2017 guidelines set by the American Academy of Sleep Medicine [21]. Harmonized scoring rules were applied, and concordance was assessed through participation in scoring proficiency testing programs as well as internal concordance testing [21]. 

OSA Severity.

The Apnoea-hypopnoea index (AHI) was calculated as the average number of apneas and hypopneas per hour of sleep. In our study, OSA was defined as an AHI of 5 or more.

AHI (total AHI, NREM AHI and REM AHI), oxygen desaturation index (ODI) and proportion of time spent with oxygen saturation below 90% (T90%) were recorded for each individual.

## Depression

We used the Depression, Anxiety and Stress Scale 21 (DASS-21) to define depression; the DASS-21 has also been validated in OSA populations [22]. 

The DASS-21 is a symptom questionnaire by Lovibond and Lovibond that contains 3 subscales which individually assess depression, anxiety and stress-related symptoms [23]. Each subscale has 7 questions and is given a score of 0 to 3. A total of 21 points can be scored on each subscale. A subscale score greater than 4 indicates depression, greater than 3 indicates anxiety, and greater than 7 indicates stress. In our study, depression was defined as DASS-21 depression subscore >4 (D >4).

## Study groups

Biobank participants were included in the study if they were (1) ≥ 18 years of age, (2) had a valid diagnostic PSG, and (3) completed a DASS-21 questionnaire.

Participants were excluded if: (1) central or mixed apneas were ≥ 50% of the total AHI); (2) Another sleep disorder was present including restless legs syndrome, narcolepsy, insomnia, or circadian rhythm sleep-wake disorder.

Participants were divided into 4 groups: (1) no OSA-no depression (AHI < 5, D ≤ 4), (2) OSA-only, (AHI ≥ 5, D ≤ 4) (3) depression-only (AHI < 5, D > 4) and (4) OSAD (AHI ≥ 5, D > 4).

## Outcome variables

Sleep architecture.

Individual data for sleep architecture including total sleep time (TST), sleep efficiency (SE), wake after sleep onset (WASO), arousal index (AI), sleep latency (SL), REM latency (REML), and proportions of NREM (NREM%), N1 (N1%), N2 (N2%), N3 (N3%), and REM (REM%) were recorded.

Quality of life scores.

Epworth Sleepiness Scale (ESS) and Functional Outcomes of Sleep Questionnaire (FOSQ-10) scores were recorded for each individual.

### Covariates

Demographic information collected on participants included age, gender, body mass index (BMI), highest education achieved, occupational status, smoking and alcohol use, and previous diagnoses of mental health disorders and sleep disorders.

### Statistical analysis

All data analyses were performed using SPSS version 29.0 software (IBM Corp, Armonk, NY, USA). Individual data was averaged to obtain grouped data for each study group, and continuous data reported as mean ± SD or median (IQR) depending on distribution. Categorical variables were compared between study groups using chi-squared test, and continuous variables using analysis of variance (ANOVA) or Kruskal-Wallis test where appropriate.

Analysis of covariance (ANCOVA) was used to examine the impact of OSA and depression on the outcomes of sleep architecture and quality of life scores. Variables which were significantly different between the study groups (*p* < 0.05) were considered as potential covariates on ANCOVA. For sleep architecture comparisons, ANCOVA was adjusted for age [24], gender [25], BMI [26], and psychiatric medications [27] due to the known influence of these variables on sleep architecture. For quality of life score comparisons, ANCOVA was adjusted for age, gender, BMI, alcohol intake and psychiatric medications. The OSA-only group was used as the reference category in the analyses.

## Results

**Fig. 1:**
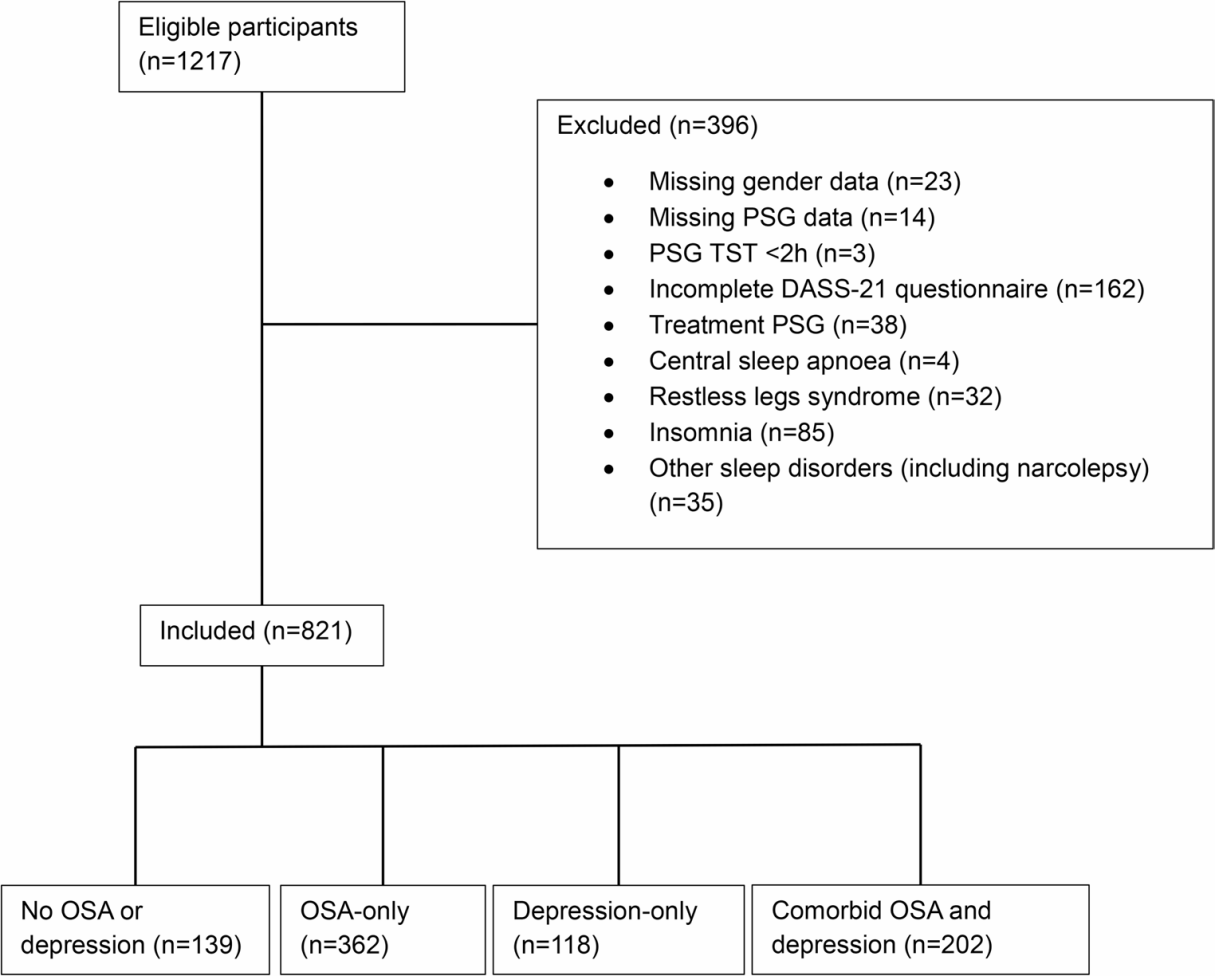
Participant selection flowchart demonstrating data included in study

There were 1217 participants who were registered in the Sydney Sleep Biobank between August 2018 and April 2023. After exclusions, 821 patients were included in the final analysis (Fig. 1).

Table 1 summarises the sociodemographic, health, and clinical variables for the study groups. There were significant differences in age, gender, BMI, smoking and alcohol consumption between groups. The depression-only group were younger with the highest proportion of women. The OSA-only group and OSAD group were older, predominantly men and more obese. A previous mood disorder diagnosis and psychiatric medication use was significantly higher in the two groups with depression, with a slightly higher proportion in the depression-only group. The most commonly used psychiatric medications were selective serotonin reuptake inhibitors (SSRIs), followed by serotonin norepinephrine reuptake inhibitors (SNRIs) (detailed breakdown of psychiatric medications can be found in Appendix 2). OSA was more severe in the OSAD group compared to the OSA-only group. AHI, ODI (3%) and T90% were all higher in the OSAD group compared to the OSA-only group.

**Table 1:** Participant characteristics by study group. (N=821)

	No OSA or Depression	OSA-only	Depression-only	Comorbid OSA and depression	*p*
**N (%)**	139 (17%)	362 (44%)	118 (14%)	202 (25%)	
**Age (yrs)**	44.6 ± 15.3	54.4 ± 14.3	38.1 ± 14.7	50.8 ± 14.1	< 0.001^†^
**Gender (%)** **Male** **Female**	67 (48%) 72 (52%)	265 (73%) 97(27%)	53 (45%) 65 (55%)	129 (64%) 73 (36%)	< 0.001^#^
**BMI kg/m**^**2**^	26.4 ± 5.8	31.6 ± 8.1	26.2 ± 5.4	33.8 ± 8.7	< 0.001^†^
**Highest education** **Primary/Secondary** **Tertiary** **Other**	34 (24%) 93 (67%) 12 (9%)	73 (20%) 240 (66%) 47 (13%)	28 (24%) 77 (65%) 13 (11%)	53 (26%) 123 (61%) 26 (13%)	0.601^#^
**Employment status** **Full-time** **Part-time** **Unemployed** **Other**	78 (56%) 20 (14%) 18 (13%) 23 (17%)	183 (51%) 52 (14%) 61 (17%) 62 (18%)	62 (52%) 21 (18%) 15 (13%) 20 (17%)	95 (47%) 29 (14%) 42 (21%) 35 (18%)	0.781^#^
**Smoking history**	40 (29%)	133 (37%)	38 (32%)	109 (54%)	< 0.001^#^
**Alcohol** **< 4 times per week** **≥ 4 times per week** **Missing**	115 (82%) 19 (14%) 5 (4%)	305 (84%) 47 (13%) 10 (3%)	136 (84%) 14 (12%) 5 (4%)	179 (89%) 20 (10%) 3 (1%)	0.019^#^
**Previous mood disorder diagnosis**	15 (11%)	43 (12%)	51 (43%)	82 (41%)	< 0.001^#^
**Any psychiatric medication use** **Antidepressant use**	18 (13%) 18 (13%)	51 (14%) 44 (12%)	34 (29%) 32 (27%)	51 (25%) 45 (22%)	< 0.001^#^
**Epworth Sleepiness Scale (ESS) a.u.**	6.7 ± 4.8	6.6 ± 4.5	9.5 ± 5.3	8.4 ± 4.6	< 0.001^†^
**Functional Outcomes of Sleep Questionnaire (FOSQ-10) a.u.**	16.0 ± 2.6	17.0 ± 2.7	12.0 ± 3.7	13.9 ± 3.2	< 0.001^†^
**AHI** **NREM AHI** **REM AHI** **ODI (3%)** **T90%**	1.6 (0.6–3.1) 1.0 (0.3–1.8) 3.0 (1.1–8.1) 0.8 (0.3–2.1) 0.0 (0.0–0.0)	19.4 (11.4–39.8) 17.1 (8.4–38.8) 28.0 (12.9–51.1) 14.1 (6.8–29.7) 0.3 (0.0-1.8)	1.2 (0.5–2.5) 0.8 (0.2–1.9) 1.6 (0.0-3.8) 0.7 (0.2–1.6) 0.0 (0.0–0.0)	25.2 (11.4–52.3) 21.4 (9.5–50.0) 33.8 (15.0-60.5) 17.1 (7.7–36.5) 0.4 (0.0-1.9)	< 0.001^‡^ < 0.001^‡^ < 0.001^‡^ < 0.001^‡^ < 0.001^‡^

Note: N = number of patients (% of the total number of patients within each study group).

Abbreviations: BMI, body mass index; AHI, apnoea-hypopnoea index; ODI, oxygen desaturation index; T90%, percentage of total sleep time spent with oxygen saturations below 90%.

*n* = 5 missing BMI, *n* = 2 missing education, *n* = 5 missing employment status.

† One-way ANOVA was used

‡ Kruskal-Wallis test was used

# Chi-square test was used

**Fig. 2:**
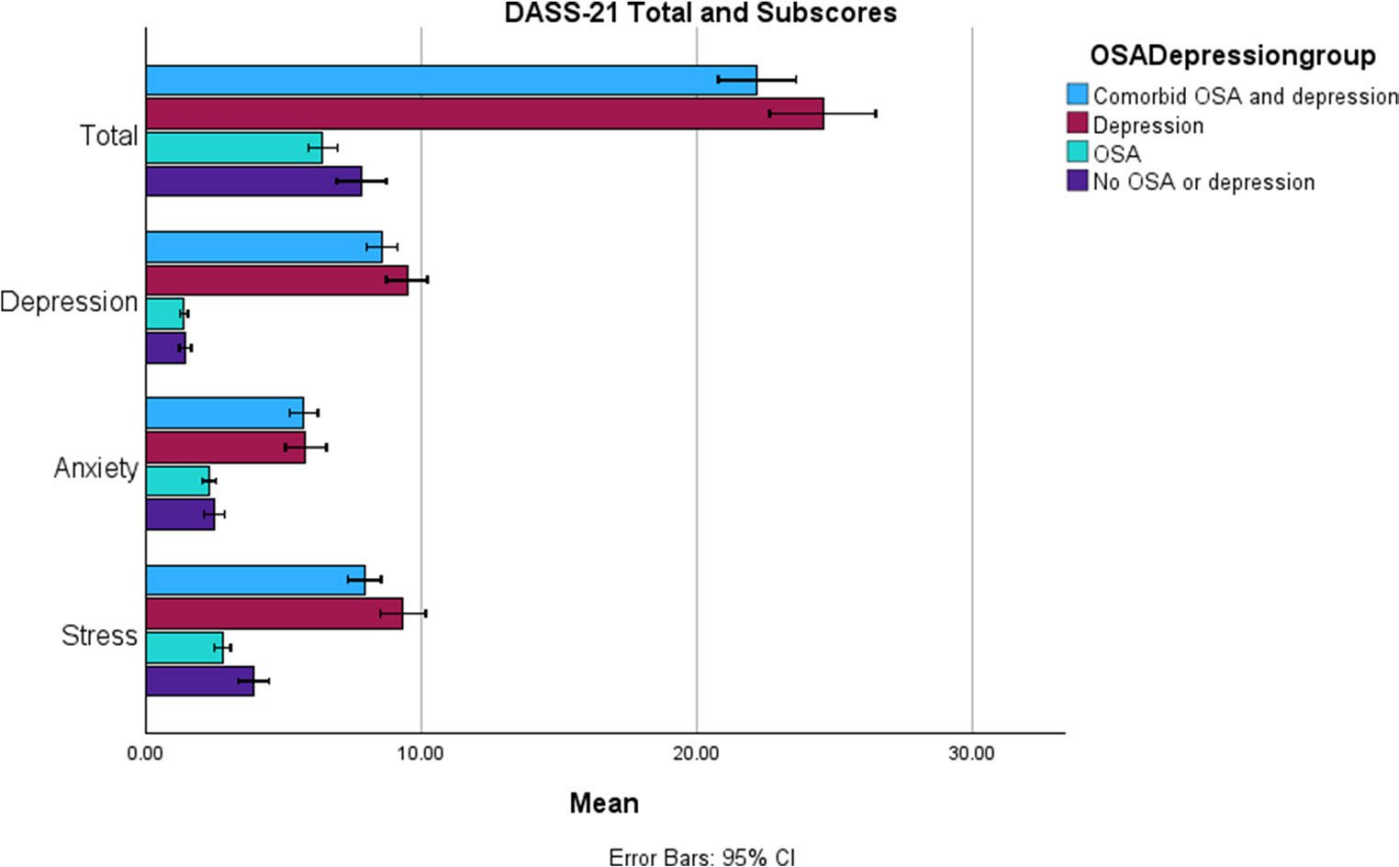
Mean total DASS-21 score and depression, anxiety and stress subscores for each of the study groups

By design, DASS-21 depression subscale scores were significantly higher in the groups with depression as compared to the groups without depression (Fig. 2). Depression and stress scores tended to be higher in the depression-only group.

### Sleep architecture

The OSAD group had the longest REM latency, highest NREM% and N1% and the lowest REM%. The groups with OSA had the highest arousal index, which was higher in the OSAD group. There were no other significant differences between groups (Table 2).

**Table 2:** Polysomnographic data by study group (N=821)

PSG Variable	No OSA or Depression	OSA-only	Depression-only	Comorbid OSA and depression	*p*
**Total sleep time (min)** **Sleep efficiency (%)** **WASO (min)** **Arousal Index**	354.6 ± 91.6 81.7 (66.1–88.1) 76.4 ± 73.1 12.6 (8.8–16.1)	345.1 ± 84.2 76.7 (66.0-85.6) 80.6 ± 59.0 27.1 (19.3–42.4)	356.9 ± 85.4 78.9 (70.4–86.6) 68.4 ± 51.5 14.7 (10.3–19.5)	337.3 ± 82.2 74.2 (65.0-83.6) 85.3 ± 58.5 30.3 (19.2–47.7)	0.141^†^ 0.013^‡^ 0.100^†^ < 0.001^‡^
**Sleep latency (min)** **REM latency (min)**	18.5 (10.5–35.5) 118.6 ± 70.2	19.1 (9.0–39.0) 121.9 ± 71.8	22.5 (13.0-42.8) 137.2 ± 80.9	21.8 (10.9–43.3) 148.2 ± 85.8	0.134^‡^ < 0.001^†^
**NREM%** **N1%** **N2%** **N3%**	82.1 ± 6.2 5.4 (3.7–8.7) 58.1 ± 10.4 17.4 ± 11.2	83.4 ± 6.6 8.4 (4.7–13.9) 55.4 ± 11.6 17.5 ± 13.0	83.7 ± 7.7 5.4 (3.4–8.3) 55.9 ± 12.3 20.2 ± 12.3	85.0 ± 7.6 9.2 (4.8–16.4) 56.3 ± 12.5 16.8 ± 11.6	0.004^†^ < 0.001^‡^ 0.156^†^ 0.098^†^
**REM%**	17.6 ± 6.3	16.4 ± 6.6	16.6 ± 7.6	14.7 ± 7.5	0.002^†^

Note: Results are displayed as mean ± SD or median (IQR)

Abbreviations: WASO, wake after sleep onset; NREM, non rapid eye movement; N1, NREM stage 1; N2, NREM stage 2; N3, NREM stage 3; REM, rapid eye movement

† One-way ANOVA test was used

‡ Kruskal-Wallis test was used

The OSA-only group had an increased arousal index, and reduced WASO and N2% compared to those without OSA and depression after adjusting for age, gender, BMI, and psychiatric medications (Table 3). When compared to the group with no OSA or depression, OSAD was only associated with an increased arousal index which was slightly higher than the OSA-only group (Table 3).

**Table 3:** Adjusted mean differences of study groups on polysomnographic outcomes

PSG variable	No OSA or depression	OSA-only	Depression-only	Comorbid OSA and depression
**Total Sleep Time (min)**	−16.8 (−39.9, 6.2)	Ref	**−29.2 (−55.0**, −3.4)*	−13.9 (−32.8, 5.1)
**Sleep Efficiency (%)**	−4.1 (−8.4, 0.2)	Ref	**−6.8 (−11.6**, −2.0)*	−2.1 (−5.7, 1.4)
**WASO (min)**	**16.9 (0.7**, 33.0)*	Ref	**21.1 (3.0**, 39.1)*	9.5 (−3.8, 22.7)
**Arousal Index (events/h)**	**−11.6 (−16.4**, −6.9)*	Ref	**−8.4 (−13.7**, −3.1)*	1.3 (−2.6, 5.2)
**Sleep Latency (min)**	−2.0 (−12.3, 8.3)	Ref	7.8 (−3.7, 19.3)	1.6 (−6.8, 10.1)
**REM latency (min)**	2.5 (−18.4, 23.5)	Ref	11.9 (−12.0, 35.7)	**17.6 (0.4**, 34.9)*
**NREM %**	0.0 (−2.0, 2.0)	Ref	1.5 (−0.8, 3.7)	1.2 (−0.5, 2.9)
**N1%**	−0.9 (−3.5, 1.7)	Ref	0.1 (−2.8, 3.0)	1.7 (−0.4, 3.9)
**N2%**	**3.6 (0.2**, 6.9)*	Ref	1.5 (−2.2, 5.3)	1.3 (−1.5, 4.0)
**N3%**	−2.4 (−5.8, 1.0)	Ref	−0.2 (−4.1, 3.6)	−1.8 (−4.6, 1.1)
**REM%**	−0.3 (−2.2, 1.6)	Ref	−1.4 (−3.5, 0.8)	−1.3 (−2.8, 0.3)

Note: Adjusted mean differences in sleep architecture variables between study groups using the group with only OSA as the reference category.

Abbreviations: WASO, wake after sleep onset; NREM, non rapid eye movement; N1, NREM stage 1; N2, NREM stage 2; N3, NREM stage 3; REM, rapid eye movement

* *p* < 0.05

When OSAD was compared against OSA-only, there was a significantly increased REML (Table 3). N1% tended to be higher (N1%: OSAD group 11.3% [95% CI 10.1%−12.6%] vs. OSA-only group 9.6% [95% CI 8.6%−10.6%], *p* = 0.173) and N3% tended to be lower (N3%: OSAD group 16.9% [95% CI 15.2%−18.5%] vs. OSA-only group 18.6% [95% CI 17.3%−019.9%], *p* = 0.594) in the OSAD group as compared to the OSA-only and depression-only groups but this was not a statistically significant difference (Table 3 and Appendix 3).

Given an increase in REML is seen in patients on psychiatric medications, especially antidepressants [28], further analysis was conducted after excluding participants from each group who were on psychiatric medications. Before exclusion, REML in the OSAD group was 17.6 min longer (95% CI 0.4–34.9 min, *p* = 0.043) than in the OSA-only group. After exclusion and adjusting for age, gender and BMI, the increase in REML was no longer significant, supporting our hypothesis that the increase in REML was driven by the effect of psychiatric medications (REM%: OSAD group 129.4 min [95% CI 118.6–140.3 min] vs. 113.5 min [95% CI 105.8–121.2 min], *p* = 0.101). There were no other significant differences in sleep architecture between OSA-only and OSAD.

### Quality of life scores

**Fig. 3:**
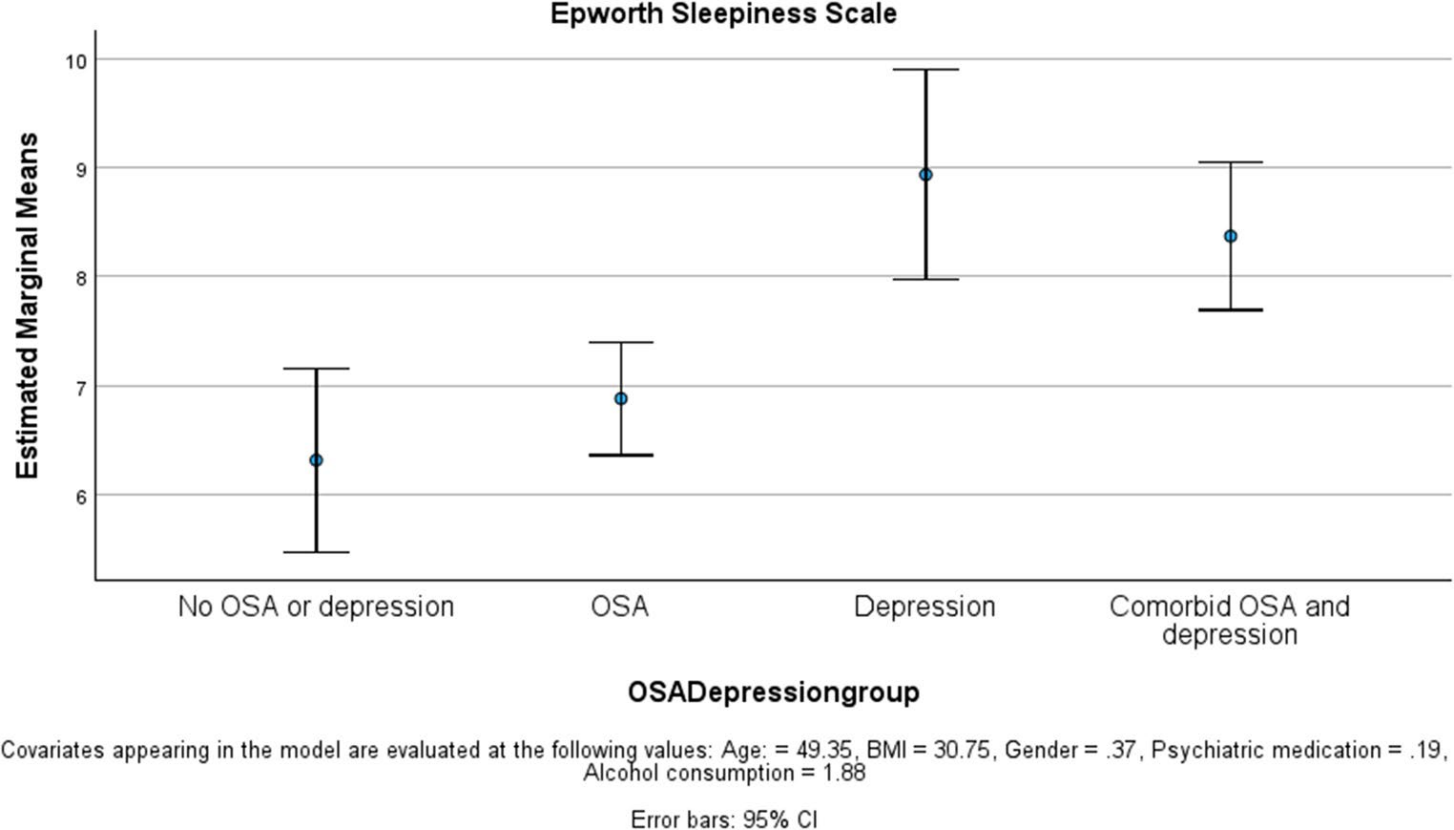
ESS difference between groups

**Fig. 4:**
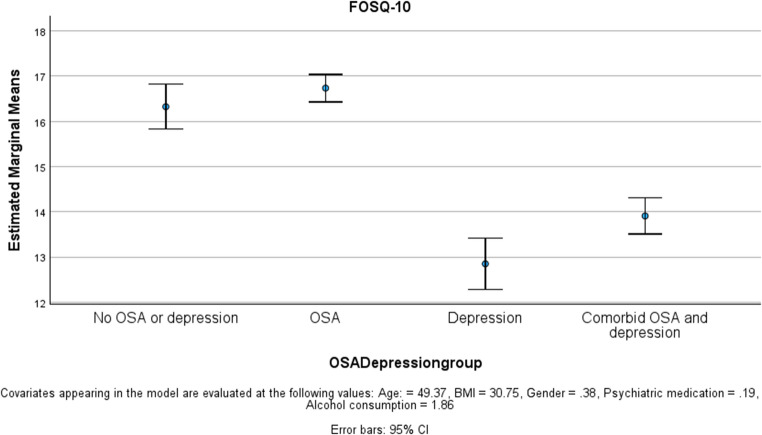
FOSQ-10 difference between groups

ESS and FOSQ-10 scores were significantly worse in the sub-groups with depression (Table 1). The depression-only group had the highest ESS and lowest FOSQ-10 scores. Whilst OSAD was associated with a lower ESS and higher FOSQ-10 compared to depression-only, this difference was not statistically significant and the ESS and FOSQ-10 scores were both significantly more abnormal than for patients with OSA-only (Figs. 3 and 4).

## Discussion

This is the largest cross-sectional study exploring differences in sleep architecture in comorbid OSA and depression in a sample of patients presenting to sleep clinics. It is also the first to use the DASS-21 as an investigative tool to define depression which has provided some insight into the associations between sleep architecture and mood disturbance. However, given the nature of the study, the ability to identify causal or temporal relationships between OSA, depression and sleep architecture is limited.

We found that comorbid OSA and depression was associated with an increased REM latency, but this effect was due to the use of psychiatric medications. Comorbid OSA and depression was also associated with worse symptoms and quality of life as shown by worse ESS and FOSQ-10 scores.

To our knowledge, only 3 previous studies have examined sleep architecture in comorbid OSA and depression [17, 18, 29]. Reynolds et al. assessed 25 male patients with diagnosed sleep apnoea between 26 and 74 years of age and found that comorbid OSA and depression was associated with increased REM latency, consistent with the current study, and reduced REM activity [17]. A study conducted by Bardwell et al. of 106 patients found that comorbid OSA and depression was associated with an increased REM% and shorter sleep latency compared to having OSA alone, neither of which was observed in the current study [18]. More recently, Wang et al.’s study reported preliminary findings in 715 patients (abstract only) referred to a Taiwanese sleep centre which did not find any significant sleep architecture differences between OSA patients with and without diagnosed depression [29]. 

The inconsistency in sleep architecture findings may be explained by the differences between studies in methodology and study population. It is now a well-established fact that psychiatric medications, especially antidepressants, suppress REM sleep [28, 30, 31]. REM suppression would expectedly both prolong REM latency and reduce REM activity. In terms of controlling for the effects of psychiatric medications to account for this, Reynolds et al. ensured all participants were free of any psychotropic medications for 14 days before undergoing PSG [17]. Despite the positive findings, they acknowledge that their findings need to be interpreted with care due to the small sample size of the study. In Bardwell and Wang’s studies, there was no control for the effects of psychiatric medications, which may have confounded their results [18, 29]. Given the relatively small number of patients in our cohort on psychiatric medications, we excluded them to attempt to gain a more accurate picture of the sleep architecture changes in relation to depression without medication. Conversely with the subset on medications, we were unable to examine the impact of different classes of psychiatric medications on sleep architecture due to the small numbers when stratifying by drug class, some participants being on more than one agent, and the lack of accurate medication dosage information.

The definitions used for OSA and depression also varied between studies which could account for some variation in sleep architecture results. Bardwell used a respiratory disturbance index (RDI) of 15 or more to define OSA [18], whereas Reynolds included patients with mixed sleep apnoea and central sleep apnoea syndrome in their study and did not define the cut-offs used for AHI or RDI [17]. Previous studies of sleep architecture stratifying groups by OSA severity have shown that more severe OSA is associated with increased N1% [32] and reduced N3% [32, 33]. The fact that we used an AHI ≥ 5 to define OSA in a select group of sleep clinic patients may also account for our inability to detect sleep architecture differences. Also, it is unclear whether central or mixed sleep apnoea causes more sleep architecture changes in adults compared with OSA-alone. We were unable to find any studies in adults describing sleep architecture changes in central sleep apnoea and mixed sleep apnoea but found a study in children which showed that concurrent central sleep apnoea with OSA reduced N3% compared to OSA-alone [34]. We excluded participants with CSA and mixed sleep apnoea from our study to eliminate confounding factors.

With regards to defining depression, Reynolds utilised the Kupfer-Detre Scale [17] and Bardwell utilised the Centre for Epidemiologic Studies Depression Scale to assess for active depressive symptoms [18], whereas Wang’s study defined participants with depression based on a clinical diagnosis [29]. Much of the literature showing that depression is associated with increased N1% and REM changes used a strict definition of depression based on either clinician-diagnosed depression [15], or fulfilment of criteria from the well-established Diagnostic and Statistical Manual of Mental Disorders (DSM), International Classification of Diseases-10 (ICD-10) [16]. Use of the DASS-21 depression subscore likely overestimated the presence of depression in our cohort as participants with depressive symptoms due to sleep disorders would score higher on the DASS-21, similar to participants with depressive symptoms due to a mental health disorder.

Our study has several limitations which may have prevented us from identifying any significant differences in sleep architecture between groups. Firstly, all participants were presenting to a sleep service with sleep complaints. This limits the external validity of this study and applicability of this information to community populations with OSA and/or depression. Furthermore, this population may have other underlying health conditions not captured by the data collection that can potentially disrupt sleep architecture. While we used OSA-only as our comparison group (rather than those with neither OSA nor depression), the clinical sample makes it harder to distinguish the four study groups on polysomnographic outcomes. Having said that, given all participants of the SSB were patients with sleep complaints, the group with neither OSA nor depression would not have been a true control group.

Secondly, we used the DASS-21 to define depression. The DASS-21 has been extensively studied and validated in many different populations, including patients with OSA, with discriminative validity for depression screening [35] similar to other established symptom scales such as the Beck Depression Inventory-II (BDI-II) [36]. In our study, 39% of all participants would have been identified as having depressive symptoms using DASS-21 depression subscores, which is in line with the prevalence of comorbid depression in OSA patients reported in other studies [5–7]. However, the DASS-21 is not a diagnostic tool for depression and does not replace the gold standard of defining depression by a standard clinician assessment using the DSM or ICD-10 criteria. As mentioned above, it does not distinguish between patients who have depressed mood due to sleep disruption and patients who have a primary mental health illness characterised by neurotransmitter imbalances. In the comorbid OSA and depression group, this would have created a heterogenous group with participants who had depressed mood caused by OSA, and participants who had both OSA and a primary depressive illness. It is also important to note that similar heterogeneity would be seen in the depression-only group, with a subset of participants with a primary depressive illness, and a subset with depressed mood due to sleep disturbance from a sleep disorder or organic illness. We found that comorbid depression was associated with poorer quality of life in participants with OSA at a level similar to participants with depression alone. This finding may be due to the fact that depression in this group represents a heterogenous condition where depression could be either due to concurrent mental illness or mood disturbance from sleep. Therefore, a clear cumulative effect between OSA and depression was not seen.

Another limitation is that we did not consider any other comorbid psychiatric conditions that may be present in this cohort. Anxiety [37], post-traumatic stress disorder [38], and schizophrenia [39] are also associated with alterations in sleep architecture which may have affected the outcomes of our study. However, we did exclude use of psychiatric medications in the analysis, which likely removed the impact of conditions that were diagnosed and treated.

## Conclusions

Comorbid OSA and depression was not associated with any significant sleep architecture differences compared to OSA alone. Higher ESS and lower FOSQ-10 scores are seen compared to OSA alone and are reflective of the worse mood experienced by this group. However, it is unclear as to whether mood disturbances in comorbid OSA and depression are due primarily to sleep disruption and nocturnal sympathetic hyperactivity from OSA, or a concurrent primary mental health disorder.

A prolonged REML may be seen in some patients with comorbid OSA and depression but is likely due to the effect of medications used in the treatment of depression. It is important for the clinician to be aware of concurrent mental illness and ensure it is adequately treated as comorbid depression can impact treatment compliance and prognosis in OSA. As such, clinicians should ask about whether patients are on psychiatric medications or have a previous mental health disorder diagnosed if a prolonged REML is seen.

Whilst the results of this study overall are negative, future studies should continue to explore the relationship between depressed mood, clinically diagnosed depression and OSA including changes in sleep architecture in response to treatment of depression and treatment of OSA. Future studies should attempt to utilise community-based cohorts with improved characterisation of mood disorders using clinician-administered structured clinical interviews for DSM disorders to obtain a more accurate picture of sleep architecture changes in mood disorders. Experimental studies utilising baseline and post-treatment PSGs should be used to examine the effects of psychiatric medications on sleep architecture. More detailed studies of sleep architecture, including sleep microarchitecture, may reveal novel changes which can identify comorbid depression in OSA patients to guide therapeutic decisions.

## Supplementary Information

Below is the link to the electronic supplementary material.

Supplementary Material 1 (DOCX 44.1 KB)

## Data Availability

The datasets analysed during the current study are available from the corresponding author on reasonable request.

## Abbreviations

PSG: Polysomnogram
TST: Total sleep time
DASS-21: Depression, anxiety and stress scale 21
OSA: Obstructive sleep apnoea

## References

[1] Senaratna CV, Perret JL, Lodge CJ, Lowe AJ, Campbell BE, Matheson MC et al (2017) Prevalence of obstructive sleep apnea in the general population: A systematic review. Sleep Med Rev 34:70–81 27568340 10.1016/j.smrv.2016.07.002

[2] Edwards C, Almeida OP, Ford AH (2020) Obstructive sleep apnea and depression: a systematic review and meta-analysis. Maturitas 142:45–54 33158487 10.1016/j.maturitas.2020.06.002

[3] Berry RBMDF, Wagner MHMDF, Ryals SMMDF Psychiatry and sleep. In: Berry RBMDF, Wagner MHMDF, Ryals SMMDF, editors. Fundamentals of Sleep Medicine2025. pp. 832–55

[4] Kerner NA, Roose SP (2016) Obstructive sleep apnea is linked to depression and cognitive impairment: evidence and potential mechanisms. Am J Geriatr Psychiatry 24(6):496–508 27139243 10.1016/j.jagp.2016.01.134 PMC5381386

[5] Harris M, Glozier N, Ratnavadivel R, Grunstein RR (2009) Obstructive sleep apnea and depression. Sleep Med Rev 13(6):437–444 19596599 10.1016/j.smrv.2009.04.001

[6] Ejaz SM, Khawaja IS, Bhatia S, Hurwitz TD (2011) Obstructive sleep apnea and depression: a review. Innov Clin Neurosci 8(8):17 21922066 PMC3173758

[7] Douglas N, Young A, Roebuck T, Ho S, Miller BR, Kee K et al (2013) Prevalence of depression in patients referred with snoring and obstructive sleep apnoea. Intern Med J 43(6):630–634 23461358 10.1111/imj.12108

[8] Wickwire EM, Cole KV, Dexter RB, Malhotra A, Cistulli PA, Sterling KL et al (2024) Depression and comorbid obstructive sleep apnea: association between positive airway pressure adherence, occurrence of self-harm events, healthcare resource utilization, and costs. J Affect Disord 349:254–261 38159653 10.1016/j.jad.2023.12.055 PMC10922426

[9] Akashiba T, Kawahara S, Akahoshi T, Omori C, Saito O, Majima T et al (2002) Relationship between quality of life and mood or depression in patients with severe obstructive sleep apnea syndrome. Chest 122(3):861–865 12226024 10.1378/chest.122.3.861

[10] Hobzova M, Prasko J, Vanek J, Ociskova M, Genzor S, Holubova M et al (2017) Depression and obstructive sleep apnea. Neuro Endocrinol Lett 38(5):343–352 29106789

[11] Liu H, Peng W, Zhou L, Shen Y, Xu B, Xie J et al (2023) Depression with obstructive sleep apnea lead to high cardiovascular disease morbidity/all-cause mortality: findings from the SHHS cohort. J Sleep Res 32(4):e13828 36732290 10.1111/jsr.13828

[12] Zhao Z, Gao Y, Lin J, Xu R, He Z, Zhao L et al (2023) Association of depression with Long-Term cardiovascular risks in older patients with obstructive sleep apnea. Nat Sci Sleep 15:1033–1043 38075392 10.2147/NSS.S423550 PMC10710184

[13] Shahveisi K, Jalali A, Moloudi MR, Moradi S, Maroufi A, Khazaie H (2018) Sleep architecture in patients with primary snoring and obstructive sleep apnea. Basic Clin Neurosci 9(2):147–156 29967674 10.29252/NIRP.BCN.9.2.147 PMC6026090

[14] Basunia M, Fahmy SA, Schmidt F, Agu C, Bhattarai B, Oke V et al (2016) Relationship of symptoms with sleep-stage abnormalities in obstructive sleep apnea-hypopnea syndrome. J Community Hosp Intern Med Perspect 6(4):32170 27609729 10.3402/jchimp.v6.32170 PMC5016742

[15] Jiang J, Li Z, Li H, Yang J, Ma X, Yan B (2024) Sleep architecture and the incidence of depressive symptoms in middle-aged and older adults: a community-based study. J Affect Disord 352:222–228 38342319 10.1016/j.jad.2024.02.020

[16] Ricciardiello A, Teh JZ, Lam AKF, Marshall NS, Naismith SL, D’Rozario AL (2024) Objective measures of sleep in adults and older adults with and without depression: a systematic review and meta-analysis. Sleep Med 124:637–648 39515262 10.1016/j.sleep.2024.10.011

[17] Reynolds CF, Kupfer DJ, McEachran AB, Taska LS, Sewitch DE, Coble PA (1984) Depressive psychopathology in male sleep apneics. J Clin Psychiatry 45(7):287–290 6735987

[18] Bardwell WA, Moore P, Ancoli-Israel S, Dimsdale JE (2000) Does obstructive sleep apnea confound sleep architecture findings in subjects with depressive symptoms? Biol Psychiatry 48(10):1001–1009 11082475 10.1016/s0006-3223(00)00887-8

[19] Amis TC, Sutherland K, Cistulli PA, Aimee L, Melehan K, Piper A et al (2019) Sydney Sleep Biobank (SSB): Development of a research resource

[20] von Elm E, Altman DG, Egger M, Pocock SJ, Gøtzsche PC, Vandenbroucke JP (2007) The strengthening the reporting of observational studies in epidemiology (STROBE) statement: guidelines for reporting observational studies. Ann Intern Med 147(8):573–577 17938396 10.7326/0003-4819-147-8-200710160-00010

[21] Berry RB, Brooks R, Gamaldo C, Harding SM, Lloyd RM, Quan SF et al (2017) AASM scoring manual updates for 2017 (Version 2.4). J Clin Sleep Med 13(5):665–666 28416048 10.5664/jcsm.6576 PMC5406946

[22] Nanthakumar S, Bucks RS, Skinner TC, Starkstein S, Hillman D, James A et al (2017) Assessment of the Depression, Anxiety, and stress scale (DASS-21) in untreated obstructive sleep apnea (OSA). Psychol Assess 29(10):1201–1209 27936819 10.1037/pas0000401

[23] Lovibond SHaL PF Manual for the Depression Anxiety Stress Scales. 2nd ed. Foundation P, editor. Sydney1995

[24] Ohayon MM, Carskadon MA, Guilleminault C, Vitiello MV (2004) Meta-analysis of quantitative sleep parameters from childhood to old age in healthy individuals: developing normative sleep values across the human lifespan. Sleep 27(7):1255–1273 15586779 10.1093/sleep/27.7.1255

[25] Krishnan V, Collop NA (2006) Gender differences in sleep disorders. Curr Opin Pulm Med. 10.1097/01.mcp.0000245705.69440.6a 17053485 10.1097/01.mcp.0000245705.69440.6a

[26] Rao MN, Blackwell T, Redline S, Stefanick ML, Ancoli-Israel S, Stone KL et al (2009) Association between sleep architecture and measures of body composition. Sleep 32(4):483–490 19413142 10.1093/sleep/32.4.483 PMC2663862

[27] Ghossoub E, Geagea L, Kobeissy F, Talih F (2021) Comparative effects of psychotropic medications on sleep architecture: a retrospective review of diagnostic polysomnography sleep parameters. Sleep Sci 14(3):236–244 35186202 10.5935/1984-0063.20200071 PMC8848521

[28] Wichniak A, Wierzbicka A, Walęcka M, Jernajczyk W (2017) Effects of antidepressants on sleep. Curr Psychiatry Rep 19(9):63 28791566 10.1007/s11920-017-0816-4 PMC5548844

[29] Wang H, Hsu W, Chang T, Chang C, Chu S (2025) Sleep architecture changes in depression and Sleep-Related disorders [Abstract]. Int J Neuropsychopharmacol 28(Supplement1):i246–i7

[30] Riemann D, Nissen C (2012) Sleep and psychotropic drugs. In: Espie CA, Morin CM (eds) Oxford handbook of sleep and sleep disorders. Oxford, Oxford

[31] Hutka P, Krivosova M, Muchova Z, Tonhajzerova I, Hamrakova A, Mlyncekova Z et al (2021) Association of sleep architecture and physiology with depressive disorder and antidepressants treatment. Int J Mol Sci 22(3):1333 33572767 10.3390/ijms22031333 PMC7866255

[32] Basunia RA, Fahmy S, Schmidt MF, Agu C, Bhattarai B, Oke V et al (2015) Sleep architecture in obstructive sleep apnea-hypopnea syndrome (OSAHS) in adult African American population and relationship with apnea hypopnea index (AHI) and Epworth sleepiness scale (ESS). Chest 148(4):354A

[33] Wu B, Cai J, Yao Y, Pan Y, Pan L, Zhang L et al (2020) Relationship between sleep architecture and severity of obstructive sleep apnea. Zhejiang Da Xue Xue Bao Yi Xue Ban 49(4):455–461 32985158 10.3785/j.issn.1008-9292.2020.08.02 PMC8800721

[34] Luo C, Chen W, Li Q (2024) The effect of central sleep apnea on sleep architecture in children with obstructive sleep apnea. Int J Pediatr Otorhinolaryngol 183:112053 39106760 10.1016/j.ijporl.2024.112053

[35] Weiss RB, Aderka IM, Lee J, Beard C, Björgvinsson T (2015) A comparison of three brief depression measures in an acute psychiatric population: CES-D-10, QIDS-SR, and DASS-21-DEP. J Psychopathol Behav Assess 37(2):217–230

[36] Gloster AT, Rhoades HM, Novy D, Klotsche J, Senior A, Kunik M et al (2008) Psychometric properties of the depression anxiety and stress scale-21 in older primary care patients. J Affect Disord 110(3):248–259 18304648 10.1016/j.jad.2008.01.023 PMC2709995

[37] Fuller KH, Waters WF, Binks PG, Anderson T (1997) Generalized anxiety and sleep architecture: a polysomnographic investigation. Sleep 20(5):370–376 9381061 10.1093/sleep/20.5.370

[38] Haberland L, Höllmer H, Schulz H, Spiegelhalder K, Gorzka R (2019) Changes in sleep architecture in German armed forces personnel with posttraumatic stress disorder compared with depressed and healthy control subjects. PLoS ONE 14(4):e0215355 30995285 10.1371/journal.pone.0215355 PMC6469790

[39] Luu B, Rodway GW, Rice E (2018) Should we be targeting sleep architecture to more effectively treat schizophrenia? JAAPA 31(12):52–54 30489392 10.1097/01.JAA.0000544303.53824.71

