# Correction to: Sleep architecture and quality of life in comorbid OSA and depression: cross-sectional analysis of the Sydney sleep biobank

**DOI:** 10.1007/s11325-026-03612-3

**Published:** 2026-02-12

**Authors:** Daryl E. C. Y. Chan, Yu Sun Bin, Philip de Chazal, Peter A. Cistulli, Andrew S. L. Chan, John R. Wheatley, Kristina Kairaitis, Brendon J. Yee

**Affiliations:** 1 Woolcock Institute of Medical Research, Macquarie University, 2 Innovation Road, Macquarie Park, Sydney, NSW 2113 Australia; 2 Sleep Research Group, Charles Perkins Centre, University of Sydney, Sydney, NSW Australia; 3 Northern Clinical School, Faculty of Medicine and Health, University of Sydney, Sydney, NSW Australia; 4 School of Biomedical Engineering, University of Sydney, Sydney, Australia; 5 Department of Respiratory and Sleep Medicine, Royal North Shore Hospital, Reserve Rd, St Leonards, NSW 2065 Australia; 6 Ludwig Engel Centre for Respiratory Research, The Westmead Institute for Medical Research, Sydney, NSW Australia; 7 Westmead Clinical School, Faculty of Medicine and Health, University of Sydney, Sydney, NSW 2145 Australia; 8 Department of Respiratory and Sleep Medicine, Westmead Hospital, Westmead, Sydney, NSW 2145 Australia; 9 Department of Respiratory and Sleep Medicine, Royal Prince Alfred Hospital, Camperdown, Sydney, NSW 2050 Australia; 10 Central Clinical School, Sydney Medical School, University of Sydney, Sydney, Australia

**Correction to: Sleep and Breathing (2025) 29:302**

**https://doi.org/10.1007/s11325-025-03485-y**

In the originally published version of this article, the name of the 7th author was incorrectly given as “Kristina Karaitis “. The correct name is “Kristina Kairaitis”, which is also given above.

Also, in the Abstract section, there are 2 spelling issues with 2 words joined together in 2 places. In the abstract conclusion, “toidentify” should be “to identify” and “worsequality” should be “worse quality”.

